# Calmodulin: A Multitasking Protein in Kv7.2 Potassium Channel Functions

**DOI:** 10.3390/biom8030057

**Published:** 2018-07-18

**Authors:** Alessandro Alaimo, Alvaro Villarroel

**Affiliations:** Instituto Biofisika, Consejo Superior de Investigaciones Científicas, CSIC, UPV/EHU, Barrio Sarriena s/n, 48940 Leioa, Spain

**Keywords:** calmodulin, calcium, potassium channels, Kv7, signal transduction

## Abstract

The ubiquitous calcium transducer calmodulin (CaM) plays a pivotal role in many cellular processes, regulating a myriad of structurally different target proteins. Indeed, it is unquestionable that CaM is the most relevant transductor of calcium signals in eukaryotic cells. During the last two decades, different studies have demonstrated that CaM mediates the modulation of several ion channels. Among others, it has been indicated that Kv7.2 channels, one of the members of the voltage gated potassium channel family that plays a critical role in brain excitability, requires CaM binding to regulate the different mechanisms that govern its functions. The purpose of this review is to provide an overview of the most recent advances in structure–function studies on the role of CaM regulation of Kv7.2 and the other members of the Kv7 family.

## 1. Calmodulin: A Ubiquitous Protein Involved in Many Different Functions

Calmodulin (CaM) is the member of EF-hand proteins superfamily which are important Ca^2+^ sensors in eukaryotic cells that have increasingly received more attention. Initially, CaM was described as an activator of cyclic nucleotide phosphodiesterases in heart and brain extracts [1,2]. However, more recently, it has been recognized for the multitasking nature and the ubiquitous localization of this Ca^2+^-binding protein that lacks enzymatic activity on its own [3]. CaM is a small (148 aa), soluble, thermostable and acidic protein ubiquitously found in animals, plants, fungi and protozoa, but absent in prokaryotic cells. In mammalians, it is widely distributed in all body tissues [4], being particularly abundant in the brain where its concentration ranges from 1 to 10 μM, or even more (~0.5% of brain proteins) [5,6]. Structurally, CaM presents two similar globular domains, the N-lobe and the C-lobe, connected to each other by a central flexible linker region. Each lobe is composed of two EF-hands, namely α-helix-loop-α-helix motifs, which are responsible for the binding of up to four Ca^2+^ ions per CaM molecule. Remarkably, the ability of CaM to interact with a large number of proteins is mostly due to its structural flexibility, particularly its’ adaptable binding surfaces [7,8].

**Figure 1:**
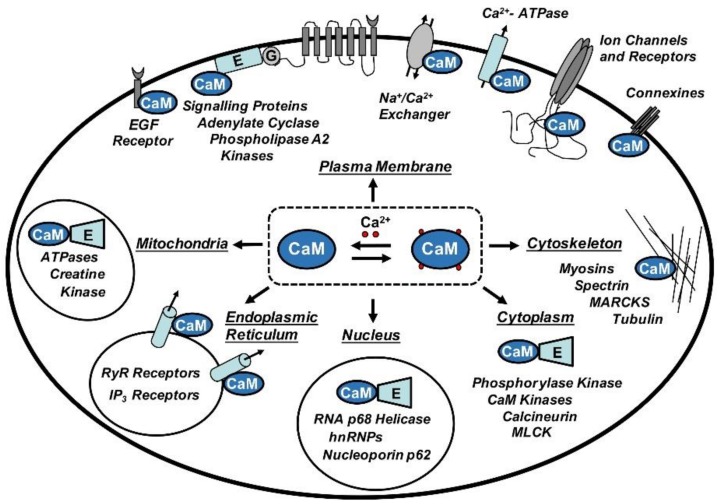
The central role played by calmodulin (CaM) in the regulation of a wide spectrum of binding partners. Abbreviations: hnRNPs: Heterogeneous nuclear ribonucleoproteins; MARCKS: Myristoylated alanine-rich C-kinase substrate; MLCK: Myosin light-chain kinase.

CaM is involved in crucial cellular pathways where it acts as a sensor or transductor of Ca^2+^ signals by regulating the function of other proteins. Among them, we find enzymes, cytoskeleton proteins, membrane transporters, receptors and ion channels implicated in a plethora of cellular functions, including inflammatory processes and immune response, smooth muscle contraction, cellular division and proliferation, gene expression, hormone and neurotransmitter secretion, apoptosis and others [9] (Figure 1).

In 1978, Brehm and Eckert described for the first time the Ca^2+^-mediated inhibition of a voltage gated ion channel in *Paramecium* [10]. Nevertheless, the molecular mechanism underlying this Ca^2+^-dependent regulation of an ion channel remained obscure until the ‘90s when it was suggested that CaM could mediate the regulation of Ca^2+^-dependent Na^+^- and K^+^-channels in *Paramecium* [11,12]. Since then, over 40 years later, several papers have demonstrated that CaM plays a pivotal role in the modulation of ion channels and receptors, such as Ca^2+^-activated K^+^ channels [13], N-methyl-D-aspartate (NMDA) glutamate receptors [14], cyclic nucleotide-gated ion channels [15], transient receptor potential (TRP) channels [16], voltage-gated Ca^2+^-, Na^+^- or K^+^-channels [17,18,19,20,21] and many others [5].

This review aims to summarize the functional findings together with the different mechanisms of action proposed for the interaction of CaM with Kv7.2 channels. Finally, we will discuss the most recent structure-function studies on the role of CaM regulation of Kv7.2 and the other members of the Kv7 family.

## 2. Multiple Roles of Calmodulin in the Regulation of Kv7.2 Channels

The human genome contains about 70 genes encoding for K^+^ channel subunits, being *de facto* the most diversified family of ion channels. Among them, *KCNQ* genes encode five Kv7 voltage-gated K^+^-channels (Kv7.1–Kv7.5). Kv7.1 is principally localized in the heart where it is responsible for the slow potassium current I_Ks_, while the other members of the family (Kv7.2–Kv7.5) are mainly expressed in the nervous system [22,23]. Kv7.2 and Kv7.3 are the main subunits of the low-threshold voltage-gated K^+^ channel termed “M-channel”, which widely regulates neuronal excitability [24,25,26,27]. Kv7 channels form homo- or hetero-tetramers and each subunit present six transmembrane segments with intracellular N- and C-terminals. Recent cryo-electron microscopy studies by MacKinnon [28] shows that the C-terminal displays four predicted α-helices (ABCD) conserved in all Kv7 family subunits [20]. Atomic structural models have revealed the existence of another helix (TW helix or post-helix A, between helices A and B). Additionally, the extended C-terminal region presents domains that are essential for the interaction with modulatory molecules, such as the membrane phospholipid phosphatidylinositol-4,5-bisphosphate (PIP_2_), and for the tetrameric assembly [29]. 

In 2002, two independent laboratories demonstrated that CaM is a binding partner of Kv7 channels [20,21]. The CaM binding domain of Kv7 channels is made up of two discontinuous sites, one located in helix A (hA) and the other in helix B (hB) [20], separated by approximately 135 residues, though another site between hA and hB in Kv7.2 (“TW helix”) could also assist in CaM binding [30]. All the members of Kv7 channels bind CaM [20], however, there is a disagreement about the Ca^2+^-dependence of this interaction. It has been proposed that Ca^2+^ does not influence the association of CaM with Kv7.2/7.3 heteromers [21], or that the interaction of Kv7.2 with CaM is weaker [31] or stronger in the absence of Ca^2+^ [20,32,33,34,35,36]. Furthermore, in vitro assays have demonstrated that apoCaM or Ca^2+^-CaM can bind peptides containing the sequences of Kv7.2 hA or hB [20,21,33,37,38,39]. In summary, it appears that the strength of the interaction of CaM with Kv7.2 channels is altered by Ca^2+^ occupancy. 

Similar to small conductance Ca^2+^-activated K^+^ channels (SK) [13], CaM was first defined as an integral subunit constitutively tethered to the C-terminal region of Kv7.2/3 channels since Kv7.2 mutants that were deficient in CaM binding were unable to generate measurable currents [5,21]. However, this model has been questioned since Kv7.2 channels, carrying a hB mutation [40] or Kv7.4 hA mutated channels [41] that do not bind CaM, can still reach the plasma membrane and are functional. In brief, it appears that the constitutive tethering of CaM is not an absolute requirement for M-channel function.

CaM is essential in the generation of functional M-current (Kv7.2/Kv7.3) in heterologous cells [37] and in neurons [42]. It is also believed to mediate the Ca^2+^-dependent inhibition [43] of Kv7.2/7.3 heterotetramers by bradykinin or UTP in sympathetic neurons [34,37,44,45]. This “Ca^2+^ sensor” function influences channel gating in a Kv7 subunit-specific manner, suppressing Kv7.2, Kv7.4 and Kv7.5 currents but stimulating Kv7.1 and *I*_KS_ channels (Kv7.1 + KCNE1) activity [46,47]. CaM binding to Kv7.1 is also required for appropriate folding of the C-terminus and it is also necessary for correct channel trafficking to the plasma membrane [47,48].

In 2007, our laboratory demonstrated for the first time the crucial role of CaM in Kv7.2 channels trafficking. We found that mutations in hA and hB underlying benign familiar neonatal convulsions (BFNC), an autosomal dominant form of neonatal epilepsy, weakened CaM binding, leading to reduced currents as a consequence of an endoplasmic reticulum (ER) retention of Kv7.2 subunits. Due to this retention, a reduced number of channels reached the plasma membrane [49]. Furthermore, we confirmed the critical role played by CaM in the intracellular transport of Kv7.2 proteins, proposing a model in which CaM needs to adopt an “active” conformation to promote the exit from the ER [32]. Subsequently, this was also observed in hippocampal neurons where CaM regulates the trafficking and the enrichment of Kv7.2/Kv7.3 channels at the axonal surface [50,51].

In the last few years, several studies have demonstrated new and intriguing roles of CaM in the regulation of Kv7.2 channels. These channels, as with all the members of the Kv7 family, necessitate phospholipid PIP_2_ for their regular function [52,53,54]. Kv7 channels are inhibited after stimulation of G_q_- and/or G_11_-protein-coupled receptors [52,55] as a consequence of the depletion of PIP_2_ upon activation of phospholipase C [56,57]. Mutations in the Kv7.2 CaM binding domain interfere with the PIP_2_ activation of the channel [57], suggesting that CaM might compete with PIP_2_ [58]. Accordingly, recent data indicate the influence of CaM in Kv.7 channels gating by producing changes in the voltage dependence of activation [59,60,61]. 

Lately, two research laboratories have provided evidence concerning the interconnection between these modulatory molecules in Kv7.2 channels function. The Naoto Hoshi’s group demonstrated that the phosphorylation of CaM, mediated by casein kinase 2, enhanced the binding with Kv7.2, induced resistance to PIP_2_ depletion, thus, leading to an augmentation on Kv7.2 current amplitude [62]. In accordance with these findings, Gomis-Perez and colleagues found that expression and availability of apoCaM alter the PIP_2_ regulation of Kv7.2 and Kv7.3 channels [60]. Another paper, performing a live-cell FRET study combined with an electrophysiological-based analysis, provided evidence of a functional connection between CaM binding, PIP_2_ dependency and the distal coiled-coil tetramerization domain in Kv7.2 channels [35]. Therefore, these data suggest that CaM affects PIP_2_ sensitivity. Finally, new observations underlined the reciprocal connection between the hAB domain and the coiled-coil module through CaM-mediated regulation of the stability of the distal tetramerization domain of Kv7.2 channels [63].

## 3. Structure-Function Studies on Calmodulin-Kv7 Channel Complexes

The Protein Data Bank (https://www.rcsb.org/) contains hundreds of structures of CaM, alone or forming complexes with different proteins. Importantly, the number of CaM structures with peptides of ion channels is still growing, advancing our understanding of CaM regulation of these membrane proteins (reviewed by [8,64]).

Concerning Kv7 channels, protein expression for structural studies, demanding huge amounts of soluble and correctly folded proteins, very often has been challenged by the poor solubility of the Kv7 proximal C-termini, resulting in insoluble and aggregated proteins. Currently, a successful approach to obtaining high quality protein samples include the elimination of disposable hydrophobic regions of Kv7 C-terminal likely to be unstructured, along with the co-expression of CaM. This strategy has produced excellent results and is consonant with the assumption that CaM may have a chaperone-like role for Kv7 family C-termini domains [47,48,65,66,67]. 

Biochemical, functional and structural studies have determined the oligomeric state of the Kv7 hAB domain-CaM complex. The results clearly indicated that CaM binds simultaneously to the hAB sites of Kv7 in a 1:1 stoichiometry. Hence, the complex is modeled as a tetramer with four subunits of Kv7.1 [48,66,68], Kv7.2 [35,67,69] or Kv7.4 [59,70] COOH-terminal and four bound molecules of CaM.

Early biochemical and functional studies have paved the way for understanding how CaM binds these channels. Thanks to a focused research effort, we know that the CaM binding domain is composed by two sites, referred to as hA and hB, which adopt an alpha helical configuration [20,68] and that the binding of CaM can take place on the same or on different subunits of the tetrameric channel [33,66]. Additionally, CaM can bind individually hA or hB, both in the presence or absence of Ca^2+^ [33]. Mruck and co-workers used an alternative approach to generate an elegant model of the Kv7.2/7.3–CaM complex [71]. In this model, the hAB-CaM complex is located at ~40 Å from the vestibule of the pore, thus, when CaM binds Ca^2+^, it can easily modulate the channel gating. Our group found that hA of Kv7.2 presents a noticeable preference for the C-lobe, while hB binds more favorably to the N-lobe of CaM [38]. Significantly, this arrangement is also displayed on the crystallographic complexes of CaM with Kv7.1 [66], Kv7.4, Kv7.5 [59] and a chimera between hA of Kv7.3 and hB of Kv7.2 [69]. As described below, this arrangement is also evident in the nuclear magnetic resonance (NMR) Kv7.2hAB-CaM complex [67] and in the cryo-EM structure of Kv7.1 [28].

**Figure 2:**
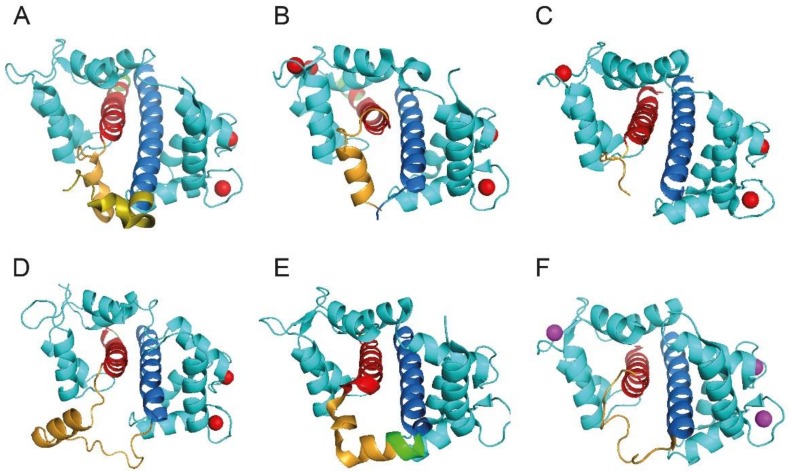
CaM/Kv7 complexes. Ribbon representation of CaM in complex with AB helices of Kv7 channels. CaM is colored turquoise, the helices A are red, the helices B are blue, the “TW domains” are in dark orange and, finally, Ca^2+^ and Mg^2+^ ions are red and magenta, respectively. Structural data were obtained from the Protein Data Bank (PDB), accession codes: (**A**) CaM/Kv 7.1hAB (4V0C, [66]); (**B**) CaM/Kv7.3hA-Kv7.2hB (5J03, [69]); (**C**) CaM/Kv7.1hAB (5VMS, [28]); (**D**) CaM/Kv7.2hAB (6FEG, [67]); (**E**) CaM/Kv7.4hAB (6B8L, [59]); (**F**) CaM/Kv7.5hAB (6B8Q, [59]). Panels A and C were simplified to show only the CaM/hAB complexes. Figures were produced with PyMOL (The PyMOL Molecular Graphics System, Version 1.5.0.4 Schrödinger, LLC.).

In the last five years, the complex formed by CaM and Kv7 family subunits has been explored using X-ray crystallography [59,66,69,70], NMR spectroscopy [67] and cryo-electron microscopy [28] (Figure 2). 

The X-ray crystallographic structure of Ca^2+^-CaM/Kv7.4 hB resembles the classical CaM 1-14 binding motif [72], with one molecule of Ca^2+^-CaM wrapping around an α-helix, namely the Kv7.4 hB segment. Likewise, in this structure, of the two CaM lobes, the N-lobe establishes more contacts with hB [70]. The results obtained by Sachyani and co-workers revealed that CaM hugs hA and hB of Kv7.1 channels, with the apo-C lobe bound to hA and the Ca^2+^-loaded N-lobe associated with hB [66]. Strikingly, this conformation is similar to that of the C-lobe of CaM-SK2 channel complex [73], which underscore structural analogies between Kv7 and SK channels. Lately, single-particle cryo-electron microscopy was used to determine the whole structure of Kv7.1 in complex with CaM [28]. Using this powerful tool, MacKinnon and colleagues have uncovered a novel interaction of CaM with the intracellular S2–S3 linker, providing evidence of an alternative functional coupling between the voltage sensor and the pore of Kv7.1 channels [28]. 

Two recent studies have determined the structures of Kv7.2-CaM complexes. Firstly, Hirsch and co-workers solved the crystal structure of the chimera Kv7.3hA-Kv7.2hB complexed with CaM at high Ca^2+^ concentration [69]. The complex displays hA bound to the CaM C-lobe, whilst Kv7.2 hB interacts principally with the N-lobe. The two helices form an antiparallel coiled-coil that resembles the Kv7.1 hAB domain described above [66]. Finally, the association of hAB domain of Kv7.2 with CaM was characterized in solution using NMR spectroscopy [67]. The authors of this study reported the structures of the CaM-Kv7.2hAB at two different Ca^2+^ concentrations: normal cytosolic Ca^2+^ concentrations (<100 nM) are able to calcify only the CaM N-lobe, while when Ca^2+^ levels increase (>1 μM), the C-lobe also can bind this cation. Precisely, the binding of Ca^2+^ to the C-lobe and the subsequent conformational rearrangement could be the structural signal responsible for modulation of channel gating. As in the previous structures, CaM wraps the Kv7.2 helices, in particular, hA binds the apoC-lobe whilst hB interacts with the calcified N-lobe. In conclusion, CaM-Kv7.2hAB structure is strikingly similar to the CaM-Kv7.1hAB, CaM-Kv7.3hA/Kv7.2hB and CaM-Kv7.4hAB or Kv7.5hAB complexes [28,59,66,69], suggesting a common, conserved CaM-mediated mechanism for transduction of Ca^2+^ signals among the members of the Kv7 family.

## 4. Conclusions

During the past 40 years, the number of ion channels found to be modulated by CaM has increased dramatically. Today, several models and accurate structures of ion channels in complex with CaM are available, improving our knowledge about the regulation of these membrane proteins.

In light of the latest progress in structure-function studies, we have summarized the most recent knowledge of Kv7 channels regulation by CaM, emphasizing numerous fundamental aspects, for which the explanation was previously hindered by the absence of structural data. In conclusion, we are confident that the recent progress on the structure-function of Kv7-CaM complexes will help to identify and evaluate the comprehensive mechanisms for mutation-induced Kv7 channel dysfunctions, such as epilepsy and long QT (LQT) syndrome.
